# School Medical and Nursing Services in South Africa

**Published:** 1920-09-04

**Authors:** 


*To the Editor of* The Hospital.

### School Medical and Nursing Services in South Africa.

*To the Editor of* The Hospital.

Sir,—Readers of the interesting account of the School Medical and Nursing Service in the Transvaal published in your issue of August 14 may like to know that a service with similar objects was organised in Natal about 1916, but, probably owing to war conditions, did not make much progress; but I understand both medical and nursing staffs are to be increased. A service was also commenced in the Cape Province at the beginning of this year, with a medical man and woman and several nurses. An inspector has also been appointed in the Orange Free State. As has happened in every other country, this work of medical inspection and the school nursing service should have large expansion in a big country like South Africa, but there are many difficulties to overcome there non-existent in older countries, such as transport, housing, etc. Medical inspection is under control of Provincial Government, and not under the Union Government. A nurse should secure a post from home before going out to Africa.—Yours faithfully,

"Colonial."

